# New Jersey State Dental Society

**Published:** 1891-02

**Authors:** 


(To be continued.)

---

### NEW JERSEY STATE DENTAL SOCIETY.—TWENTIETH ANNUAL SESSION.

(Continued from vol. xi., page 782.)

*Dr. Welch.*—So much has been stated in the paper and in the discussion which followed it that it does not leave much room for anything further to be said on the subject. The conclusion of the essayist has been that we are evidently coming to a state of thoughtfulness in the profession and not mere practice; and it is a very good feature. We all of us have good thoughts, but only a few of us use them. We all have some genius, but there are so few of us who know how to take the thoughts of genius as they pass. Therefore there are only a few successful dentists in the highest sense of that term. There are a great many who are mere mechanics and do things because they have learned to so do them, and because they have been taught by others; while there are others who are thoughtful and progressive.

So it is in public education. Very much more good could be done than is done to educate the public if dentists would impart to their patients the knowledge which would be of benefit to them while under their control. Our mouths are usually shut to such things while we work away with our instruments and show ourselves simply as mechanics. A mere word passed by a dentist to a patient in the proper way and at the proper time often sows the seeds of thought for a whole lifetime, and sometimes for the lives of the children afterwards. While we should all strive to be skilful operators, we should also seek to be popular educators; and while we are skilful with our fingers, there should also be an activity of thought and promptness in using opportunities. In this way we command a position that is desirable. It will be our own fault if we do not do so.

*Professor C. N. Peirce.*—I have just learned that the original paper on this subject, which I did not have the pleasure of hearing, was rather suggestive of the education of the public as aiding in skilful operation. Am I correct?

*The President.*—Yes, sir; you are.

*Professor Peirce.*—I would like to say a word or two on the subject. I think that very much can be done for our patients by the appropriate use of a few words at the proper time. An instance occurred of that kind in my own practice. A lady brought a child of about fourteen years of age to me for examination. After examining the mouth I said to the mother that the child was not in the habit of using her teeth, but that the food was washed into the stomach without proper mastication. I came to that conclusion from the appearance of the teeth and gums. I said, "I presume your child does not spend more than ten or fifteen minutes at her meals at a time." She replied, "That is the truth; she washes everything into her stomach with milk and water." I told the mother it would be folly for me to attempt to treat the child's teeth until some change was made in her habits of life, and that if she would take her home and deprive her of any liquid during mealtime for three months, I was satisfied the teeth would not suffer during that time, and that then I would do what I could to save them. The mother was conscientious in the treatment. She took the child home, and assured me that during the period I mentioned and until she came to me again she took her meals without the use of liquids, the liquid being taken before and after meals. I could not tell you of the improvement in that mouth during those three months,—in the cleanliness and appearance of the teeth. All but the inferior incisor teeth needed filling, and by insisting that the child should eat her food as dry as possible the teeth were kept well; but I am satisfied that had not the instructions been given the fillings would not have saved them, and that by the time she was twenty-one or twenty-two she would have been almost toothless with the exception of the inferior incisors. I base my judgment entirely upon what I believe is true, and that is, that use always aids in health and development. Disuse is a predisposing cause of decay in the teeth. In my practice I have always advised my patients, where they had teeth decaying rapidly, to eat their food as dry as possible, to masticate it thoroughly, and to give the teeth as much use as they could, and that little advice to my patients has been of great assistance to me and of very great advantage to the patients themselves.

*Dr. J. A. Osmun.*—In closing the discussion I have not anything to add. The discussion has simply carried out my ideas in a better way than I did. The idea I had in speaking of the higher education of dentists in manual training was to illustrate the fact that many men have good memories and can answer many questions on an examination and yet know very little indeed concerning the subject. In my experience I have had to do with the graduates of three or four different colleges, who actually knew nothing at all about the practical work they were called upon to perform,—the execution was entirely at fault. That is the reason I took the position I did on that point. I have no desire to lower in any way the standard of theory; raise it as high as you will,—the higher the better. Still, I do not think that is enough; we should have plenty of manual dexterity as well.

My plan for the education of the masses has been touched upon very ably by the gentlemen who participated in the discussion. I claim I am right in that. There is the utmost ignorance in the vast majority of the people at large, and the only ones who have been educated at all in dental matters are the patients of those who have been self-sacrificing enough to take some time to explain to them the different operations. We constantly meet people who are intelligent in every other direction, but wofully ignorant concerning the care of their own mouths. It is not an uncommon experience for people to come in and say, "I would like to have this child's tooth extracted." On examination the tooth is found to be a permanent one, but your visitor will argue with you, saying the child had never had a tooth taken out there, and insist that it is a first tooth. These are not ignorant people in general matters, and the only reason they lack knowledge in this direction is because nothing is ever seen in the daily papers in the way of teaching the general public upon this subject. That is the reason why I called upon the dental editors and secretaries of dental societies to disseminate these matters. We ought to give them out, but we keep them to ourselves. We have meetings where papers are read full of pregnant thoughts useful to the public. We have a duty to perform in educating the people as well as in educating ourselves, and when we fail to do it we are not living up to the highest standard.

The endowment of dental colleges, that has been spoken of, is one that lies very near my heart. I hope to see fewer colleges, and those so well supplied with means that they can be—like Cæsar's wife—above suspicion.

In reference to physicians and our relations with them, I read a paper, I think, before this society on that very subject. Physicians do not know all about dentistry any more than we do about medicine. I am happy to say that in my own city the physicians are very courteous and kind in sending patients who have any trouble in the oral cavity to the dentist, to see what their difficulty is.

I am very thankful to the gentlemen who followed me for their very courteous treatment of my paper, and to the other members of this society for the very kind way in which they received it.

On motion, the paper was passed.

On motion, the three applications for membership were referred to the Board of Examiners.

### *Wednesday, July 16, 1890.—Evening Session.*

Secretary called the roll.

Committee on President's Address reported progress.

The following persons were proposed for membership: Edward B. Frost, D.D.S., of Elizabeth, N. J.; Henry Pfheiffer, D.D.S., of Newark, N. J.; William L. Fish, D.D.S., of Roseville, N. J.

The Examining Board reported favorably upon the applications for membership of A. S. Kniffen, D.D.S., of Trenton, N. J.; D. W.

Kleinhaus, D.D.S., of Newark, N. J.; B. F. Tillyer, D.D.S., Dover, N. J.; and R. H. Sheppard, D.D.S., of Phillipsburg, N. J.

Dr. William H. Trueman then read a paper on "Erosion."

(For Dr. Trueman's paper, see page 86.)

DISCUSSION.

*Professor James Truman.*—The subject of this paper, it seems to me, is so largely suggestive rather than demonstrative of any particular course of procedure that I am left in the position of being forced to define what I regard as erosion and abrasion as preliminary to the consideration of the general subject.

I take the position that erosion and abrasion are extremely simple things, governed by a certain law of chemical action, and that there is really but trifling difference between the two conditions. Abrasion is caused, necessarily and primarily, by attrition with antagonizing teeth and aided, possibly, in all cases by acid action. Erosion is the result, unquestionably, of chemical solution and the added effect of wear of lips and particles of food. It is impossible, in my opinion, to separate these two conditions.

It is true that abrasion may occur through long attrition in mastication, and that the acid action is so insidious that it is, ordinarily, not considered as a factor in the destruction. It is, therefore, fair to conclude that we have two processes at work,—one, the wearing away by attrition, and the other softening the dental structure by acids.

When erosion proper is considered, the labial and buccal surfaces, usually of the superior teeth, are found to be destroyed and presenting peculiar manifestations of destructive action. The result is, however, not markedly different from that which occurs on the masticating surface through abrasion, and I cannot possibly draw a line between these two pathological processes.

Teeth thus acted upon are extremely dense teeth. It does not happen in early childhood, but is observed after the individual has reached a period of maturity and the teeth have become very dense.

The process which operates to produce caries on the labial surfaces of the superior incisor teeth in a child is the same in kind modified by age and conditions. In a child of eight to fifteen it rapidly progresses to so-called "white decay," as the influences, at this period, are on an entirely different structure incapable of similar manifestations to that of teeth at fifty.

Caries and erosion must be broadly distinguished from each other, as they have only a moderate degree of similarity. Erosion is a chemical action upon the tooth-structure, while caries, although produced by chemical solution, has other factors entering into combination to produce destructive results. The fact that a slight irritation, as the wearing down of the anterior teeth, produces an extra development of tissue is well known to every one present. This led me long ago to formulate this in this way,—that slight irritation produced increased development of tissue, and that over excitation produced destruction.

I now come to the consideration of the practical points of this paper of Dr. William Trueman, in which I think he has scarcely done me justice. It is one thing to furnish a clue to the solution of a vexed problem, and quite another thing to make subsequent investigations from the foundation laid. The question of erosion is one that has interested me for a series of years, as it was one of the problems for which no explanation had been given.

From a long series of tests and examinations, I had found that the secretions of the mouth during the daytime generally gave a neutral response. From that result I came to the conclusion that this question to be solved required more extended examination and in different directions. Recognizing the fact that the secretions giving a neutral response were in a condition to rapidly change to acid under proper surroundings, the effort was made to discover when and where this was likely to occur. The fact that secretions in localities where but slight motion of fluids were present generally presented a slight acid reaction led to the hypothesis that possibly the fluids of the mouth at night might be found to be increasingly acid. The difficulty of making any extended investigations here was apparent; but the work was begun on my own person. The oral secretions were carefully tested during the day without result. A further test immediately on awakening gave a marked acid reaction. This was so clear and positive that it naturally pointed to the cause of erosion. This was followed by other tests in mouths affected by erosion and in young mouths with affected labial surfaces, until the evidence accumulated and was of such a pronounced character that I did not pretend to doubt that the largest amount of destruction of teeth through caries and erosion occurred at night. From these initial observations others took up the subject, and my class of students aided me materially by corroborative results. Since that period a few others have become interested, and have, as in the case of my friend Dr. Kirk, carried the solution of the problem a step farther.

I wish to say here that these facts, narrated in substance at the discussion of Dr. Darby's paper on erosion, read before the Odontological Society of Pennsylvania, were not given by way of suggestion, but as facts demonstrated. Dr. Kirk was present at that meeting, and it was through this statement that he was led to make his admirable investigations and produce the satisfactory results. His conclusions, however, were all based on the facts I presented in regard to periods of rest and subsequent fermentation. While I am speaking I have no doubt but that there is a change going on in my own mouth, and that the secretions would exhibit a more or less acid reaction were they tested. The dryness of the mouth indicates a cessation of the flow of saliva, and its absence increases acidity. During the night this condition is generally maintained for hours. The lips are held in close contact with the labial surfaces of the anterior teeth, and there must be a chemical solution constantly transpiring.

What is true of these teeth is equally and increasingly true of all cavities of decay and of all confined places. Now, all this is very simple to my mind, and I should have been pleased if my friend, Dr. William Trueman, had given the subject, as far as the origin of this hypothesis is concerned, a clearer statement. I do not wish to stand on this question in the light of a doubter. I have no question as to the main facts. I have been obliged to speak of my personal connection with this, as it is important for the elucidation of the subject, though unpleasant in other respects.

Investigation in this matter, in my opinion, has two prominent features about it, one is the chemical and the other the clinical. This division may and probably does cover most of the investigations we, as dentists, are called upon to perform. These two modes may be regarded as the positive and the negative of all these forms of examination, and the policy of using one without the other is not to be commended, yet it is in the knowledge of every one that clinical experience has solved many problems in advance of chemical demonstration.

The essayist stated in the paper that fillings placed in cavities caused by erosion were of no avail, or, in his own language, he could not see how they could preserve a tooth under such conditions. It certainly would be impossible; but it is rare indeed that a filling can be placed in such teeth without a great sacrifice of tooth-tissue, and then conditions must be entirely changed to expect ultimate success.

Serious objections have been made to the chemical theory stated on account of the high polish presented in all cases of erosion. Those of you who remember the old narrow clasp in use many years ago,—half-round wire,—will recall the fact that this clasp wore into the tooth, and the dentine became very dense and highly polished. This condition of the surface was, without doubt, caused by the constant movement of the clasp and the passage of fluids back and forth. The destruction of tissue was caused by the retention of the secretions against the tooth, eventually causing the slow but certain solution of tooth-material.

The statement of the essayist, in his final conclusion in regard to erosion, is that acid action is simply a tradition. It is more than a tradition, as it took its place along with the chemico-vital theory of a past generation. At present it has passed beyond speculation and has reached the positiveness of a demonstration, and I cannot understand why other explanations of this process are given in view of my original and the subsequent work of Dr. Kirk.

At the time when I suppose Dr. Trueman was preparing this paper presented to you to-day, he sent me a patient with the request that I would give an opinion and suggest a remedy for the superior incisor teeth, very extensively eroded. I examined the teeth of the young man, and informed him, as I have you, that the trouble was the result of acid action during the night. I tested his oral secretions and found, as I anticipated, a neutral response. His reply to my conclusions was not complimentary, and asserted his lack of faith in any such theory. He finally, however, consented to follow my instructions in regard to testing with litmus paper.

In a few days I received a letter from him acknowledging his error, and stating that he had tested the oral secretions early in the morning, and enclosed me the paper as evidence that my diagnosis was correct. I have these slips and will now show the decided evidence of acid action.

Now, a word as to treatment. That, necessarily, must be very simple, an antacid being all that is required in connection with constant care as to cleanliness. This very simplicity causes some to reject it. If I am correct in my conclusions, an agent must be sought for that will neutralize acidity for the period named during sleep, and there is nothing better in my opinion than one of the forms of prepared chalk. The advantage of this is that it will remain on the teeth longer than other agents if applied in a thick magma. This course I have invariably adopted in young children, where there is a tendency to destruction of labial surfaces through green stain or direct action of acid and with most satisfactory results. There is sometimes a tendency to gastric disturbance if too long continued; but this is readily recognized and the treatment intermitted. The same course must be followed in erosion in both cases, remembering that mere day treatment will be of no value, but that the teeth must be carefully attended to during the twenty-four hours if good results are to be secured.

I have a case now under care, the most serious presentation of erosion it has been my privilege to examine, as it involves all the anterior teeth to fully half their depth in an antero-posterior direction. I saw the case some six years ago for the first time. Filling was out of the question, so I placed him under the treatment described, and he has faithfully followed it without, as far as I can observe, any further loss of tooth-tissue.

To my mind the evidence is sufficient, and we must look for the cause of erosion in the change of the secretions of the mouth, that it is altogether chemical, and the high polish is the result of the combined action of the lips, the passing of fluid and particles of food.

